# A prospective pilot study on DACHAO intervention for improving embryo quality metrics in IVF/ICSI failure cases

**DOI:** 10.3389/fcell.2026.1746912

**Published:** 2026-02-04

**Authors:** Rui Xing, Lei Jiang, Jie Zhang, Xiao-Qi Zuo, Xin Xu, Meng-Chi Xue, Yi-Ran Zhao, Gui-Min Hao

**Affiliations:** 1 Department of Reproductive Medicine, The Second Hospital of Hebei Medical University, Shijiazhuang, China; 2 Hebei Center for Quality Control and Management of Human Assisted Reproductive Technology, Shijiazhuang, China; 3 Hebei Key Laboratory of Infertility and Heredity, Shijiazhuang, China

**Keywords:** 2PN rate, DACHAO reco18, embryo quality, implantation rate, IVF/ICSI, pregnancy rate

## Abstract

**Research question:**

Embryo quality serves as a pivotal determinant in assisted reproductive technology (ART) outcomes, with high-grade embryos correlating with improved pregnancy/live birth rates but poor morphology elevating the risks of implantation failure and pregnancy loss. Despite its clinical significance, therapeutic options for enhancing embryo development remain limited, necessitating novel interventions to improve poor embryogenesis.

**Objective:**

This study aimed to evaluate the efficacy of DACHAO Reco18—a formulation containing natural extracts of clove, Sophora flower bud, and yam—in improving reproductive outcomes among IVF patients with a history of poor embryo quality.

**Design:**

This was a prospective self-controlled study, to assess the effects of DACHAO Reco18 containing natural extracts of clove, Sophora flower bud, and yam on reproductive outcomes in 68 IVF patients (aged 20–40 years) with a history of IVF/ICSI failure and documented poor embryo quality (≤20% high-grade embryos and no surplus embryos). All participants had previously undergone IVF/ICSI treatment at our center between January 2022 and December 2023. Over 6 weeks of oral treatment, outcomes were compared between pre- and post-intervention cycle. The primary outcome measure was embryo parameters, while secondary outcomes encompassed oocyte yield, fertilization efficiency, implantation rate and pregnancy rate.

**Results:**

DACHAO Reco18 intervention significantly improved the mean oocyte yield from 7.955 to 8.955 oocytes retrieved per cycle, fertilization outcomes with 2 pronucleus (PN) zygotes increasing from 3.731 to 5.463 embryos, and Day-3 embryo enhancement from 1.896 to 3.000 viable embryos. High-quality embryos surged by 118.52% from 0.567 to 1.239, with the quality rate improving from 30.729% to 44.107%. Additionally, when compared to the pre-treatment cycle, the post-treatment cycle showed statistically significant improvements in the implantation rate (0%–32.00%), biochemical pregnancy (0%–24.00%) and clinical pregnancy (0%–21.05%). Subgroup analyses revealed FSH reduction in hyper-gonadotropic patients (from 12.083 to 9.941 mIU/mL).

**Conclusion:**

This prospective self-controlled study suggests that a six-week DACHAO Reco18 intervention is associated with improved ovarian response and embryological outcomes in IVF patients with poor embryo quality, including reduced FSH in hyper-gonadotropic individuals. While promising, these preliminary findings warrant verification through randomized controlled trials to evaluate its potential as an adjuvant treatment in ART.

## Introduction

*In vitro* fertilization and embryo transfer (IVF-ET) remains the primary treatment approach for infertility, but with poor success rates despite advancements in ovulation induction and laboratory techniques (Alpha Scientists in Reproductive Medicine and ESHRE Special Interest Group of EmbryologyESHRE Special Interest Group Embryology, 2011a; Lundin et al., 2023). Embryo quality has been identified as the most critical determinant of IVF outcomes. High-quality embryos can significantly improve the pregnancy and live birth rates (Günther et al., 2022; Sun et al., 2020; Wei et al., 2020; Zou et al., 2023), while poor-quality embryos increase the risk of implantation failure, miscarriage (Xia et al., 2020), and ectopic pregnancy (Anzhel et al., 2022). This holds true even in natural cycle IVF, where embryo quality remains paramount for successful implantation (Tomic et al., 2020). Poor embryo quality remains a major challenge in the assisted reproductive technology (ART), significantly affecting the rate of implantation and live birth. Multiple factors compromise embryo development, such as chromosomal abnormalities associated with advanced maternal age, cellular damage induced by oxidative stress, mitochondrial dysfunction, and impaired cytoplasmic maturation. Additional contributing factors include suboptimal culture conditions, including, but not limited to, deviations in the pH value, suboptimal oxygen tension, and nutrient imbalances, as well as epigenetic alterations arising from hormonal imbalances or exposure to environmental toxins. Moreover, high levels of sperm DNA fragmentation and oocyte spindle defects may further impair fertilization and early embryogenesis.

Given the multifactorial nature of poor embryo quality, comprehensive interventions targeting multiple pathological pathways - including antioxidant, anti-inflammatory, and mitochondrial-supportive strategies - are crucial for high-risk patients. While current approaches predominantly focus on extrinsic interventions such as advanced embryo selection (via time-lapse imaging and AI algorithms) and laboratory optimization (culture media composition and oxygen concentration control) (Rienzi et al., 2011; Hill et al., 1989; Cimadomo et al., 2023; Busnelli et al., 2021), intrinsic factors - particularly genetic abnormalities, mitochondrial dysfunction, and oxidative stress - continue to present major obstacles to optimal embryo development. The development of adjuvant therapies to enhance embryo quality has therefore become a research priority. DACHAO, a commercially available maternal and preconception nutritional supplement with over 3 years of market presence, is formulated from clove ethanol extract, Sophora flower buds, and yam in the ratio of 15:6:10. Researchers have demonstrated its therapeutic potential—in aged mouse models, with the formulation having restored ovarian function through multiple mechanisms including oxidative stress mitigation, anti-inflammatory effects, mitochondrial membrane potential improvement, and serum estradiol elevation (Liu et al., 2022). Its anti-apoptotic properties were evidenced by downregulation of cleaved caspase-3 and PARP, while reduced pro-inflammatory chemokine levels confirmed anti-inflammatory efficacy (Liu et al., 2022). These multi-target mechanisms suggest DACHAO’s promise as a novel intervention for improving human reproductive outcomes.

Our interest in this product stems not only from its robust preclinical efficacy but also from its favorable safety profile as a naturally derived, food-grade botanical extract. Moreover, its oral administration route facilitates prospective clinical research. We therefore conducted a prospective clinical study to evaluate whether DACHAO could improve outcomes in women with prior IVF/ICSI (intracytoplasmic sperm injection) failure and poor embryo quality.

## Materials and methods

### Study design and implementation

**FIGURE 1:**
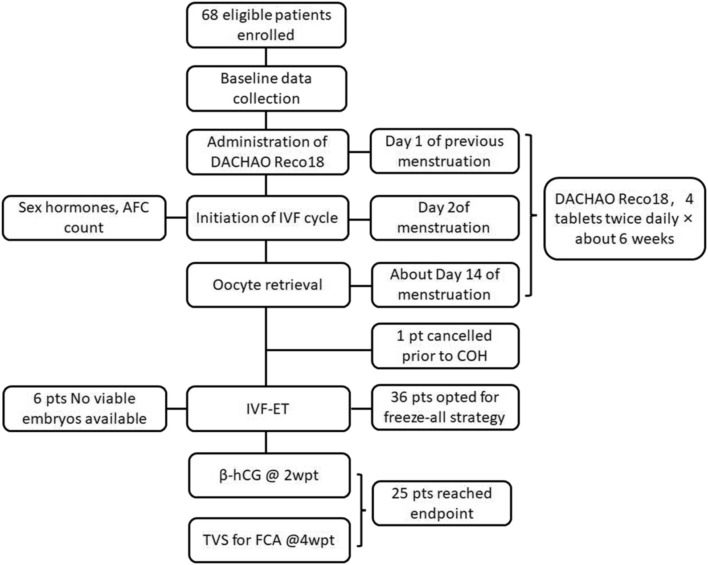
Flowchart of patient enrollment.

This prospective self-controlled study (ChiCTR2400083368) was conducted in our hospital between May 2024 and February 2025 after written informed consent obtained from the participants. All participants had previously undergone IVF/ICSI treatment at our center between January 2022 and December 2023. The current intervention cycle of these participants was used as the observation group while the previous IVF/ICSI cycles of these participants were used as the control. The inclusion criteria were women with a history of poor embryo quality (≤20% high-quality embryo rate in prior IVF/ICSI cycles with no transferable embryos), aged 20–40 years (reproductive age), body mass index (BMI) 18–24 kg/m^2^ (normal range), and indications for IVF/ICSI according to the ESHRE guidelines (ESHRE Guideline Group on RPL, 2018) (Figure 1). The exclusion criteria were women with recurrent implantation failure ≥3 failed transfers with high-quality embryos without clinical pregnancy, moderate-to-severe endometriosis (r-AFS stage III–IV with a score ≥16), untreated hydrosalpinx confirmed by ultrasound without prior laparoscopic intervention, active endometrial pathology, including polyps or chronic endometritis (diagnosed via hysteroscopy), unsuitable for assisted reproductive technology (ART) procedures, conditions incompatible with pregnancy, pathogenic chromosomal abnormalities confirmed by karyotype analysis, recurrent spontaneous abortion (RSA), preimplantation genetic testing (PGT), and drugs administered within 30 days before enrollment.

### Intervention protocol

#### DACHAO Reco18 administration

DACHAO Reco18 is a commercially available maternal and preconception nutritional supplement. Each 500 mg capsule contains a mixture of clove extract, Sophora flower bud extract, and Chinese yam extract in a mass ratio of 15:6:1. The content of eugenol in the clove extract was 90%, the content of quercetin in the Sophora flower bud extract was 95%, and the content of diosgenin in the Chinese yam extract was 15%. All the active contents had been qualified by high performance liquid chromatography (Supplementary Figure S1A). The chemical structural formulas of these bioactive compounds are displayed in Supplementary Figure S1B–D. Thus, in every 100 mg of DACHAO, the content of eugenol, quercetin and diosgenin is 43.55 mg, 18.39 mg, and 4.84 mg, respectively.

Participants underwent a 6-week oral DACHAO Reco18 pretreatment prior to repeat ovarian stimulation, with the dosage 4 tablets twice daily (total daily dose: 4000 mg), starting from the first day of the previous menstrual period (PMP) until the day of egg retrieval (approximately 6 weeks).

#### IVF/ICSI-ET treatment procedures

The gonadotropin-releasing hormone (GnRH) antagonist protocol was the first-line choice for most participants. Luteal phase GnRH agonist protocol or early-follicular phase GnRH agonist (GnRH-a) long protocol was performed in some participants. Moreover, to isolate the effect of the intervention, the same controlled ovarian hyperstimulation (COH) protocol was employed for all participants in both the pre- and post-treatment cycles. The starting gonadotropin dose in the post-treatment cycle was matched to the final effective dose from the patient’s own pre-treatment cycle to ensure comparability. All other aspects of monitoring, triggering, and laboratory procedures were kept consistent between cycles.

The GnRH antagonist protocol: Flexible GnRH antagonist protocol was used in this study. When the leading follicle was observed to be ≥ 14 mm in diameter or estradiol concentration reached ≥400 pg/mL, GnRH-antagonist (0.25 mg/day, Merck Serono, Coinsins, Switzerland) injection was started until the trigger day. When three dominant follicles reached 17 mm in diameter, the final maturation of oocytes was induced by recombinant hCG (250 ug; Merck Serono, Coinsins, Switzerland). The oocyte aspiration was performed 36.5 h after triggering.

Luteal phase GnRH-a protocol: A short-acting GnRH-a (Triptorelin, Ferring AG, Germany) was administrated daily in the mid luteal phase of the preceding cycle. Fourteen days later, follicular ultrasonography, serum LH, FSH and E2 were examined and 150–300IU recombinant follicle-stimulating hormone (FSH) and/or urinary FSH was used daily when FSH and LH was <5 IU/L and E2 was <50 pg/mL. GnRH-a was continued until trigger. When three dominant follicles reached 17 mm in diameter, the final maturation of oocytes was induced by recombinant hCG (250 ug; Merck Serono, Coinsins, Switzerland). The oocyte aspiration was performed 36.5 h after triggering.

Early-follicular phase GnRH agonist long protocol: Patients received an injection of triptorelin acetate (3.75 mg; Ferring Pharmaceuticals, Kiel, Germany) on 2–4 days of menstrual cycle and COS started 28–35 days after the injection. When at least three dominant follicles reached the diameter of 18–20 mm, recombinant hCG (250 ug; Merck Serono, Coinsins, Switzerland) was injection intramuscularly. The oocytes aspiration was performed 36.5–37.5 h after trigger.

Freeze-all strategy was implemented in patients with high risk of ovarian hyperstimulation syndrome (OHSS) and high progesterone levels on the trigger day. Fresh embryo transfer was performed on day 3–6 after oocyte retrieval, depending on the number of embryos and patients’ clinical history. The luteal phase support was performed by vaginal administration of progesterone gel (Crinone, Merck Serono, Watford, United Kingdom) starting from the morning of oocyte retrieval day until 14 days after ET.

#### Embryo assessment and grading

All oocyte retrievals, fertilization procedures and embryo cultures were performed in the same IVF laboratory under identical conditions and by the same embryology team, using standardized media and protocols.

All embryos were evaluated according to the comprehensive Istanbul Consensus criteria throughout development. The oocytes were inseminated approximately4 to 6 h after oocyte retrieval by a conventional method or intracytoplasmic sperm injection. Fertilization was assessed at 16–18 h post-insemination by the presence of two pronuclei (2PN). Day 2 embryos (44 ± 1 h post-insemination) and day 3 embryos (68 ± 1 h post-insemination) were classified according to the blastomere number, symmetry, and fragmentation percentage (Alpha Scientists in Reproductive Medicine and ESHRE Special I nterest Group of Embryology, 2011b). Cleavage-stage embryos (days3 post-fertilization) were classified as good-quality when demonstrating: 1) 7-9 blastomeres on Day 3, 2) equal-sized blastomeres (<10% variation), 3) ≤10% fragmentation, and 4) absence of multinucleation.

Blastocyst embryos (116 ± 2 h post-insemination) were classified according to the stage of development, the inner cell mass (ICM) and trophectoderm (TE) (Alpha Scientists in Reproductive Medicine and ESHRE Special I nterest Group of Embryology, 2011b; Gardner et al., 2000). The blastocysts assessment and grading criteria was as follow: Blastocysts were given a numerical score from 1 to 6 on the basis of their degree of expansion and hatching status, as follows: 1, an early blastocyst with a blastocoel that is less than half of the volume of the embryo; 2, a blastocyst with a blastocoel that is half of or greater than half of the volume of the embryo; 3, a full blastocyst with a blastocoel completely filling the embryo; 4, an expanded blastocyst with a blastocoel volume larger than that of the early embryo, with a thinning zona; 5, a hatching blastocyst with the trophectoderm starting to herniate though the zona; and 6, a hatched blastocyst, in which the blastocyst has completely escaped from the zona. For blastocysts graded as 3–6 (i.e., full blastocysts onward), the development of the ICM was assessed as follows: A, tightly packed, many cells; B, loosely grouped, several cells; or C, very few cells. The TE was assessed as follows: A, many cells forming a cohesive epithelium; B, few cells forming a loose epithelium; or C, very few large cells. All assessments were performed by two experienced embryologists, with discrepancies resolved by a third senior embryologist.

For cleavage embryos, Grade 1 embryos were classified as good-quality embryos (Alpha Scientists in Reproductive Medicine and ESHRE Special I nterest Group of Embryology, 2011a); for blastocysts, a grade ≥ 3BB (including AA, AB, BA, and BB) was defined as good-quality embryos (Gardner et al., 2000).

### Outcome measurement

Various variables and outcome measurement were examined, and the baseline demographic data were collected for each patient, including female age, body mass index (BMI), infertility cause, hormonal levels and AMH levels in both ovarian stimulating cycles.

The primary endpoint was the good-quality embryo numbers, and the secondary endpoints were total oocytes retrieved for the ovarian response, 2PN fertilization rate for the fertilization efficiency, total number of transferable embryos (including frozen embryos), serial measurements of FSH, AMH, and E_2_ before and after intervention for the endocrine dynamics, absolute number of high-quality embryos yielded per cycle, early biochemical pregnancy rate (β-hCG >25 IU/L at 14 days post-transfer), implantation rate, clinical pregnancy (observation of an intrauterine gestational sac on transvaginal ultrasound or villous tissue confirmed by histology).

The rate calculations were as follows: Good-quality embryo rate = number of good-quality embryos at Day 3/total number of embryos at Day 3; 2PN fertilization rate = the number of oocytes observed with 2PN/the total number of oocytes fertilized by ICSI/IVF; available embryo rate = number of available embryos/number of normal fertilized embryos.

### Statistical analysis

Statistical analyses were performed using the SPSS version 29.0 (SPSS, Inc., Chicago, IL, United States) and R language. The study analyzed the baseline demographic characteristics and compared the clinical parameters and reproductive outcomes between two consecutive IVF/ICSI cycles among the 68 enrolled patients. The statistical tests confirmed that the data in this study were predominantly non-normally distributed. For quantitative indicators of therapeutic effects (including FSH, AMH, and E2 levels; number of oocytes retrieved, 2PN zygotes, total usable embryos, high-quality embryos, and high-quality embryo rate), the Wilcoxon signed-rank test (paired non-parametric test) was used, with results expressed as median (M) and interquartile range (IQR, 25th–75th percentiles). For categorical indicators (including biochemical pregnancy rate, clinical pregnancy rate, embryo implantation rate, and live birth rate), comparisons were made using the Chi-square test, with data presented as numbers and percentages. The significant P value was set at <0.05.

## Results

### Subjects

Sixty-eight patients were enrolled who met the following criteria: previous failed IVF/ICSI treatment, high-quality embryo rate ≤20%, and no remaining transferable embryos (Table 1).

**TABLE 1:** Clinical characteristics of couples enrolled in this study.

Variables		Data (n = 68)
Female age (years)		34 (30,36)
BMI (kg/m^2^)		23.2 (21.7,25.0)
Infertility cause (n,%)	Female factor	72.1 (49)
	Male factor	16.2 (11)
	Both female and male factors	2.9 (2)
	Unexplained	8.8 (6)
Infertility type	Primary (n,%)	57.4 (39)
	Secondary (n,%)	42.6 (29)
Infertility duration (years)		3 (1,5.5)
Failed ET cycles		1 (1,2)
Hormonal level of previous ovulation induction	FSH	5.73 (4.90, 7.52)
	LH	3.77 (2.57,5.11)
	E2	31.18 (22.50, 50.69)
	P	0.54 (0.33,0.89)
	PRL	13.36 (9.30,19.23)
	T	0.38 (0.31,0.50)
	AMH level of previous ovulation induction	1.45 (0.65, 3.99)

Continuous variables were presented as Median (Quartile 1, Quartile 3). Categorized variables were presented as % (n). BMI, body mass index; ET, embryo transfer; FSH, Follicle-stimulating hormone; LH, luteinizing hormone; E2, estradiol; P, progesterone; PRL, prolactin; T, testosterone; AMH, Anti-Müllerian Hormone.

### Parameters of the ovarian stimulation between the pre- and post-treatment cycles

The key ovarian stimulation parameters between the pre- and post-treatment cycles were shown in Table 2. The COH protocols remained identical between the two cycles. Moreover, no significant alterations in total Gn dosage, Gn duration, E2 levels and endometrial thickness on hCG day were observed between the pre- and post-treatment cycles.

**TABLE 2:** Comparison of the ovarian stimulation parameters between the pre- and post-treatment cycles.

Variables		Pre-treatment cycle		Post-treatment cycle		P
COH protocol (n, %)	GnRH antagonist	51 (75.00%)		51 (75.00%)		​
	Luteal phase GnRH agonist	1 (1.47%)		1 (1.47%)		
	Early-follicular phase GnRH agonist long protocol	16 (23.53%)		16 (23.53%)		
Variables		Median (Q1, Q3)	Average	Median (Q1, Q3)	Average	​
Total Gn dosage (IU)		2250.00 (1800.00 2831.25)	2320.03	2400.00 (2043.75 3000.00)	2580.68	0.068
Gn duration (day)		9 (8 10)	9.07	9 (8 11)	9.63	0.293
E2 levels on hCG day (pg/mL)		1503.00 (874.25 2764.85)	1902.69	1600.9 (910.25 3705.35)	2165.42	0.313
Endometrial thickness on hCG day (mm)		10.00 (9.08 12.00)	10.89	10.00 (8.78 11.45)	10.3	0.184

Continuous variables were presented as Median (Quartile 1, Quartile 3). COH, controlled ovarian hyperstimulation; GnRH, gonadotropin-releasing hormone; Gn, gonadotropin; E2, estradiol; hCG, human chorionic gonadotropin.

### Effects on ovarian reserve biomarkers

**FIGURE 2:**
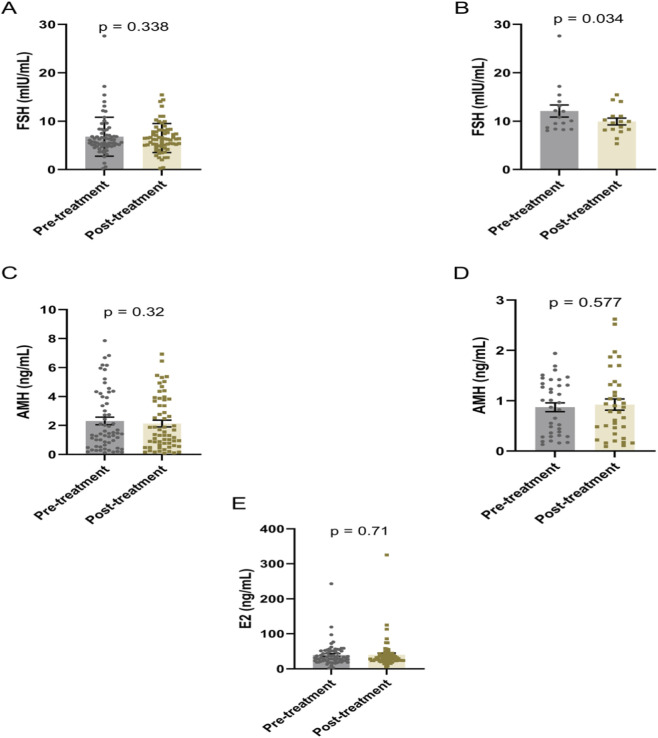
Effects of DACHAO Reco18 administration on FSH, AMH, and E2 levels in patients. **(A)** DACHAO Reco18 showed no significant regulatory effect on FSH levels in the overall patient population. **(B)** DACHAO Reco18 reduced FSH levels in patients with elevated baseline FSH (>8 mIU/mL). **(C)** DACHAO Reco18 did not significantly affect AMH levels in the overall cohort. **(D)** DACHAO Reco18 showed no significant effect on AMH levels in patients with low baseline AMH (<2 ng/mL). **(E)** DACHAO Reco18 had no significant impact on E_2_ levels.

The DACHAO Reco18 exerted differential regulatory effects on ovarian reserve biomarkers across distinct patient subgroups (Table 3). Although no significant alterations in FSH, AMH, or E_2_ levels were observed in the general study population (p ≥ 0.05) (Figures 2A,C,E), subgroup analysis revealed clinically relevant effects in specific cohorts: patients with baseline FSH >8 mIU/mL exhibited a statistically significant reduction in FSH levels post-treatment (12.083 versus 9.941, p = 0.034), suggesting therapeutic potential in hypergonadotropic conditions (Figure 2B). Notably, this FSH-lowering effect was not accompanied by concurrent changes in AMH (even in patients with baseline AMH <2 ng/mL) or E_2_ levels (all p ≥ 0.05), suggesting a targeted modulation of pituitary-ovarian feedback rather than direct ovarian stimulation (Figures 2D,E). These differential responses suggest that DACHAO Reco18 may primarily enhance ovarian sensitivity to FSH, rather than directly augmenting ovarian follicular reserve or steroidogenesis.

**TABLE 3:** Effects of DACHAO Reco18 administration on FSH, AMH, and E2 levels in patients.

Variables	Pre-treatment		Post-treatment		P
	Median (Q1, Q3)	Average	Median (Q1, Q3)	Average	
FSH (total, mIU/mL) (n = 68)	5.73 (4.90, 7.52)	6.80	6.28 (4.91, 8.09)	6.54	0.34
FSH (>8 mIU/mL) (n = 16)	10.44 (8.46, 13.80)	12.08	9.80 (8.23, 11.00)	9.94	0.03
AMH (total, ng/mL) (n = 61)	1.45 (0.65, 3.99)	2.34	1.38 (0.66, 3.77)	2.11	0.32
AMH (<2 ng/mL) (n = 36)	0.96 (0.31, 1.34)	0.88	0.87 (0.46, 1.32)	0.92	0.58
E2 (ng/mL) (n = 68)	31.18 (22.50, 50.69)	39.27	31.42 (23.63,39.01)	39.59	0.71

Continuous variables were presented as Median (Quartile 1, Quartile 3). FSH, Follicle-stimulating hormone; AMH, Anti-Müllerian Hormone; E2, estradiol.

### Improvement effects on oocyte yield, fertilization rate, and clinical pregnancy outcomes in IVF cycles

**FIGURE 3:**
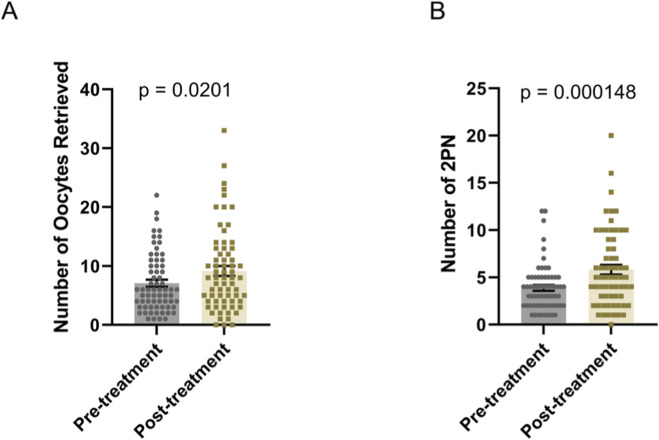
Effects of DACHAO Reco18 supplementation on oocyte yield and fertilization rate. **(A)** DACHAO Reco18 administration significantly increased the total number of retrieved oocytes. **(B)** DACHAO Reco18 supplementation improved the number of fertilized 2PN oocytes.

After oral administration of DACHAO reco18, patients underwent another *in vitro* fertilization (IVF) cycle using either an antagonist protocol or a luteal-phase short-acting long protocol. DACHAO reco18 effectively improved the total oocyte yield from 7.955 to 8.955 oocytes compared to the previous cycle (p < 0.05), indicating its efficacy in enhancing ovarian response and follicular recruitment (Figure 3A; Table 4). This finding is consistent with the observed reduction in basal FSH levels among patients with FSH>8 mIU/mL. Following conventional IVF, the 2PN fertilization rate was also significantly increased from 3.731 to 5.463 zygotes (p < 0.05), confirming that DACHAO reco18 improves the oocyte fertilization competence (Figure 3B; Table 4).

**TABLE 4:** Effects of DACHAO Reco18 supplementation on oocyte yield, fertilization rate.

Variable	Pre-treatment (n = 68)		Post-treatment (n = 68)		Z	P
	Median (Q1, Q3)	Average	Median (Q1, Q3)	Average		
No. of oocytes retrieved (n = 67)	6.00 (3.00, 10.00)	7.955	7.00 (4.00, 12.00)	8.96	2.18	0.0295
No. of 2PN(n = 67)	3.00 (2.00, 5.00)	3.731	5.00 (2.00, 8.00)	5.46	3.53	0.0004

Continuous variables were presented as Median (Quartile 1, Quartile 3). PN, pronucleus.

### Improvement on embryo development and reproductive outcome

**FIGURE 4:**
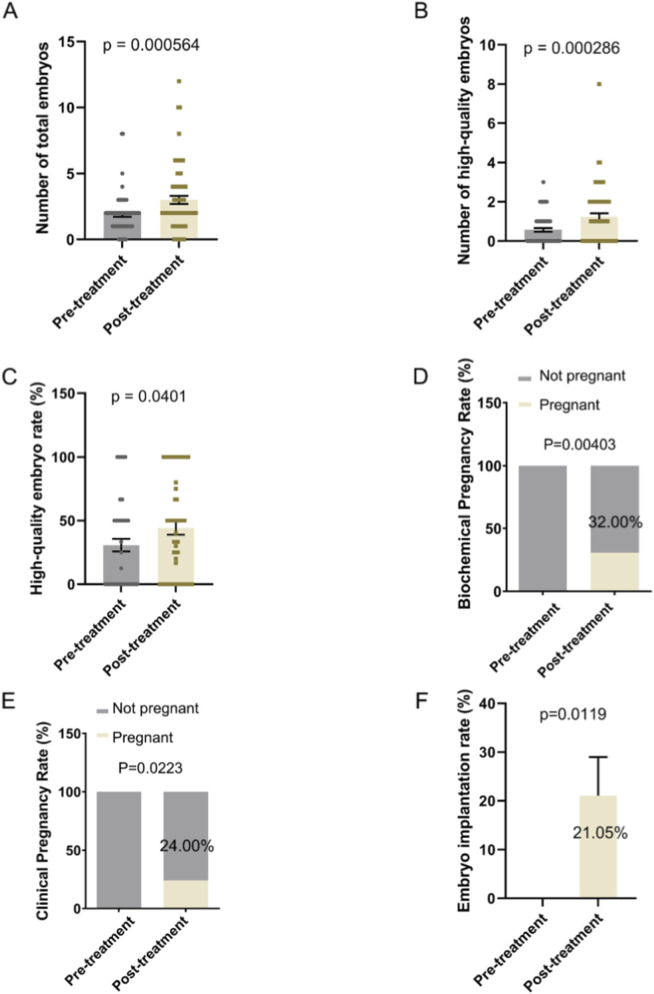
Effects of Dachereco18 (DACHAO) supplementation on total embryo count, high-quality embryo number, high-quality embryo rate and reproductive outcome. **(A)** Dachereco18 administration significantly increased the total embryo count in patients. **(B)** Dachereco18 administration improved the number of high-quality embryos in patients. **(C)** Dachereco18 administration enhanced the high-quality embryo rate in patients. **(D)** DACHAO Reco18 treatment enhanced the biochemical pregnancy rates. **(E)** DACHAO Reco18 treatment enhanced the clinical pregnancy rate. **(F)** DACHAO Reco18 treatment enhanced the embryo implantation rate.

Oral supplementation with DACHAO Reco18 significantly improved embryo development outcomes in IVF patients, with the total day-3 embryo count increased from 1.896 to 3.000 compared to their previous treatment cycle (p < 0.05), demonstrating enhanced oocyte developmental potential (Figure 4A; Table 5). Furthermore, the high-quality embryo count increased remarkably from 0.567 to 1.239 (p < 0.05), while the high-quality embryo rate rose from 30.729% to 44.107% (p < 0.05, Figures 4B,C; Table 5). These results indicate that DACHAO supplementation not only enhances embryo quantity but also significantly increases embryo quality, suggesting a positive impact on ART outcomes. Finally, serum β-hCG levels were measured 2 weeks post-transfer, and the biochemical pregnancy rate following the first fresh embryo transfer cycle was significantly improved from 0% to 32.00% (p < 0.05). Additionally, at 4 weeks post-transfer, ultrasound-confirmed gestational sacs with cardiac activity were used to determine the clinical pregnancy rate, which demonstrated a significant increase from 0% to 24.00% (p < 0.05) over baseline. Additionally, following DACHAO Reco18 treatment, the embryo implantation rate in patients reached 21.05%. These findings further support that DACHAO Reco18 may have a positive effect on embryo implantation potential and early pregnancy outcomes (Figures 4D,E).

**TABLE 5:** DACHAO Reco18 supplementation improves embryo development.

Variables	Pre-treatment (n = 68)		Post-treatment (n = 68)		Z	P
	Median (Q1, Q3)	Average	Median (Q1, Q3)	Average		
No. of total embryos (n = 67)	2 (1, 2)	1.896	2 (2, 4)	3.00	3.45	0.0006
No. of high-quality embryos (n = 67)	0 (0, 1)	0.567	1 (0, 2)	1.24	3.63	0.0003
High-quality embryo rate (%) (n = 67)	0.00 (0.00, 50. 00)	30.729	45.00 (0.00, 95.00)	44.11	2.05	0.041

Continuous variables were presented as Median (Quartile 1, Quartile 3). No., number.

## Discussion

This prospective, self-controlled clinical study investigated the potential of a novel intervention to address poor embryo quality—a significant challenge in the management of female infertility—among IVF-ET patients with a documented history of suboptimal embryological outcomes. Oral supplementation with DACHAO Reco18 for 6 weeks prior to a subsequent treatment cycle was associated with improvements across multiple intermediate endpoints. These included the number of oocytes retrieved, fertilization rates, embryo quality and yield, as well as rates of biochemical pregnancy, clinical pregnancy, and embryo implantation.

Poor embryo quality represents a critical challenge in assisted reproductive technology and typically results from a combination of intrinsic and extrinsic factors. These include advanced maternal age, oxidative stress, mitochondrial dysfunction, and suboptimal follicular microenvironment. Age-related meiotic incompetence in oocytes often leads to chromosomal abnormalities that significantly impair blastocyst formation. Furthermore, excessive reactive oxygen species (ROS) not only disrupt oocyte maturation and early embryonic development, but the accompanying decline in ovarian reserve also exacerbates gonadotropin resistance, further compromising embryo viability.

Clinically, several methods have been used to better embryo quality. Growth hormone supplementation has been shown to improve the embryo quality in poor responders (Yovich and Stanger, 2010; Tesari et al., 2005), primarily through promoting follicular development and oocyte quality. This effect may be mediated by increased FSH/LH receptor expression in granulosa cells (Yovich and Stanger, 2010; Yovich et al., 2019). Importantly, our study demonstrated that DACHAO Reco18 lowered the basal FSH levels while increasing the embryo yield, suggesting similar modes of action. Additionally, antioxidants such as melatonin and coenzyme Q10 have been shown to improve oocyte yield and high-quality embryo rates while reducing gonadotropin dosage requirements (Shang et al., 2024; Yong et al., 2021). In previous studies, CoQ10 pretreatment improved ovarian response to stimulation and embryological parameters in young women with poor ovarian reserve, while in women over 35 years it also improved oxidative metabolism in follicular fluid and oocyte quality (Xu et al., 2018; Giannubilo et al., 2018). It was confirmed that exogenous melatonin, which scavenges ROS via the Sirt1/SOD2 pathway, improved oocyte quality and suppressed aneuploidy both *in vitro* and *in vivo* (Zhang et al., 2020). Given its rich content of antioxidant compounds including quercetin, DACHAO Reco18 may confer benefits through comparable antioxidant mechanisms. Thus, DACHAO Reco18 may improve both oocyte yield and embryonic developmental potential/quality in women undergoing assisted reproductive technology cycles by simultaneously reducing ovarian hyper-responsiveness and oxidative stress levels. Dehydroepiandrosterone (DHEA) is a steroid secreted from ovarian theca cells that induce testosterone and estradiol biosynthesis (Burger, 2002), which could enhance gonadotropin responsiveness (Dewailly et al., 2016). A meta-analysis including 1,336 DHEA pretreatment cycles demonstrated that adding DHEA could increase ovarian response in women with DOR but cannot improve clinical pregnancy and live birth rates (Conforti et al., 2025). Myo-Inositol (myo-ins) is an insulin-sensitizing natural molecule that facilitates cellular glucose uptake. Recent studies have demonstrated a positive correlation between elevated levels of myo-Ins in human follicular fluid and satisfactory oocyte quality (Dinicola et al., 2021). A meta-analysis including 935 women with myo-Ins pretreatment concluded that the use of myo-Ins significantly improved the clinical pregnancy rate (p = 0.03), with a significantly reduced abortion rate (p = 0.0006) and a significant increase in the number of grade I embryos (p = 0.02). At the same time, the FSH dosage of participants with myo-Ins was significantly less (p = 0.004) (Zheng et al., 2017).

As we all know, the complete follicular physiology from the primordial to the preovulatory stage spans approximately 90 days. In many studies, the duration of adjuvant treatment lasted for 3 months (Zhang et al., 2014; Kara et al., 2014). To the best of our knowledge, there were few studies designed to explore the duration of adjuvant treatment resulting in differences on IVF outcome (Giannubilo et al., 2018; Feng et al., 2023). A recent meta-analysis hypothesized that there might be a dose- and time-dependent relationship between different GH protocols and IVF outcomes (Shang et al., 2022). According to the high time cost, the pretreatment durations of adjuvant supplements (e.g., CoQ10, DHEA) ranged from 1 to 3 months in many studies (Xu et al., 2018; Conforti et al., 2025; Aragona et al., 2025). A shorter, targeted intervention period (6 weeks) improves patient adherence and facilitates more efficient participant recruitment.

In animal studies, DACHAO was found to improve the fertility in aged mice via several mechanisms, both by *in vitro* fertilization and natural mating (Liu et al., 2022). DACHAO improves oocyte quality in aged female mice both qualitatively and quantitatively. In addition, DACHAO administration in aged female mice improved the quality of embryos and increased the growth rate of their offspring. The mechanisms were as follow: Firstly, DACHAO administration promoted the proliferation of the granulosa cells around the follicles and reduced the level of apoptosis in the ovaries. Furthermore, DACHAO administration improved the hormone level and increased the expressions of antioxidant factors in aged female mice. Additionally, the mRNA expression levels of ovarian inflammation-related factors in the mice with DACHAO administration were downregulated (Liu et al., 2022).

As a content of the DACHAO, eugenol was shown to improve tissue damage and oxidative stress after ovarian torsion in adult female rats (Barghi et al., 2021). A previous study demonstrated that clove extract enhanced blastocyst development in frozen mouse oocytes derived from in vitro-fertilized embryos (Bahmanpour et al., 2018). Moreover, several *in vitro* studies on animal and human granulosa cells showed that quercetin treatment reduced the proportion of early apoptotic cells, improved oocyte quality, and promoted subsequent embryo development (Wang et al., 2017; Rashidi et al., 2019; Silva et al., 2018). Furthermore, in vitro maturation of oocytes from humans and aged mice, quercetin subsequently improves the quality of oocytes, promoting both oocyte maturation and early embryonic development in humans and aged mice oocytes (Cao et al., 2020). A novel protein, DOI, isolated from Chinese yam was reported to stimulate estradiol biosynthesis in rat ovarian granulosa cells, with a potential to treat menopausal syndrome (Wong et al., 2015).

We observed a statistically significant reduction in FSH levels. Importantly, this effect was not accompanied by changes in AMH or E_2_, suggesting a targeted modulation of the pituitary-ovarian axis rather than direct ovarian stimulation. This subgroup represents a clear and mechanistically supported target population, as lowering FSH in hypergonadotropic women may improve follicular sensitivity and oocyte quality.

This prospective, self-controlled study provides preliminary clinical evidence suggesting that oral supplementation with DACHAO Reco18 may be associated with improved embryological parameters and early pregnancy outcomes in a selected cohort of IVF patients with a documented history of poor embryonic development. Compared to their own previous untreated cycles, participants undergoing the intervention demonstrated notable improvements in key intermediate endpoints, including oocyte yield, fertilization efficiency, and notably, the number and proportion of high-quality embryos. An increase in clinical pregnancy rates was also observed following treatment. The observed associations, though statistically significant, do not establish causality, as potential confounding from inter-cycle variability cannot be entirely ruled out. Nonetheless, the consistent pattern of improvement across multiple developmental stages lends biological plausibility to the notion that the multi-component botanical formulation in DACHAO Reco18 could positively influence the follicular and embryonic microenvironment. This provides a rational basis for future investigation. The primary clinical implication of this pilot study is to justify and inform the design of a larger, randomized, placebo-controlled trial.

Several study limitations should be acknowledged. The relatively small sample size may limit the generalizability of findings, and the limited number of embryo transfer procedures reduced the statistical power of pregnancy outcomes analysis. While significant improvements were observed in implantation and clinical pregnancy rates, this study was not specifically powered for these clinical endpoints due to the sample size. The pregnancy outcomes should therefore be interpreted as promising preliminary signals that require confirmation in larger, adequately powered trials. In addition, the pre-post, self-controlled study design, though appropriate for a preliminary investigation in this refractory population, inherently limits the ability to attribute outcomes solely to the intervention due to potential inter-cycle variability and the absence of a parallel control group. Moreover, enrollment of Chinese patient, no randomization, and no involvement of multiple centers may also produce some publication bias to affect the outcomes and generalization of the study. Future randomized, double-blind, placebo-controlled trial (RCT) with involvement of a larger cohort of patients and multiple centers will be necessary to conclusively establish the clinical value of DACHAO Reco18 supplementation for IVF patients with poor embryo quality.

## Conclusion

In conclusion, this prospective self-controlled study indicates that a six-week intervention with DACHAO Reco18 was associated with significant improvements in ovarian response and embryological parameters among IVF patients with a history of poor embryo quality. A reduction in FSH levels was also observed in the hyper-gonadotropic subgroup. While these findings are encouraging, the self-controlled design precludes definitive causal inferences and cannot fully account for inter-cycle variability. Therefore, the results should be interpreted as preliminary evidence supporting the need for further research. Future randomized, placebo-controlled trials are warranted to rigorously evaluate the efficacy and potential role of this botanical formulation as an adjuvant treatment for ART patients with poor embryo quality and compromised pregnancy prognosis.

## Data Availability

The original contributions presented in the study are included in the article/Supplementary Material, further inquiries can be directed to the corresponding author.
