# Supplementary material for: A prospective pilot study on DACHAO intervention for improving embryo quality metrics in IVF/ICSI failure cases
Source: Front Cell Dev Biol. 2026 Feb 4;14:1746912. doi: 10.3389/fcell.2026.1746912 (PMC12913368; doi:10.3389/fcell.2026.1746912)
Supplement: Supplementary file 1 [file DataSheet1.docx]

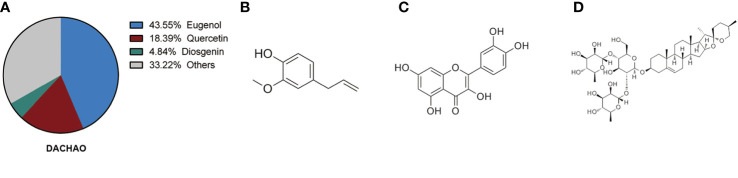


Supplementary Figure 1. DACHAO’s main components. (A) Via high performance liquid chromatography test and calculation, the proportion of each main component in DACHAO. (C–D) Chemical structures of eugenol, quercetin, and diosgenin.
